# Menarche as a silent transition: islamic modesty norms, mother–daughter communication, and menstrual readiness among rural Indonesian adolescents

**DOI:** 10.3389/frph.2026.1820596

**Published:** 2026-06-11

**Authors:** Yulli Fety, Dewi Sari Pratiwi, Asri Dwi Novianti

**Affiliations:** Bachelor of Nursing and Professional Nurse Program, Faculty of Health Sciences, Universitas Mandala Waluya, Kota Kendari, Southeast Sulawesi, Indonesia

**Keywords:** islamic modesty norms, menarche, menstrual readiness, mother–daughter communication, rural adolescents

## Abstract

**Background:**

Menarche is a key developmental milestone for adolescent girls, yet in many Muslim-majority rural settings it occurs within strong norms of modesty and silence. Although prior research has identified gaps in menstrual knowledge and hygiene, limited evidence explains how Islamic modesty norms and mother–daughter communication jointly influence menstrual readiness during the early transition around menarche.

**Objective:**

This study examined menarche as a culturally patterned silent transition by exploring (1) how Islamic modesty norms shape adolescents’ silence and disclosure practices, (2) the association between mother–daughter communication quality and menstrual readiness, and (3) implications for culturally safe nursing, school, and community interventions for rural Indonesian adolescents.

**Methods:**

An explanatory sequential mixed-methods design was applied. The quantitative phase involved a school-based survey of 386 Muslim girls aged 10–15 years in rural Indonesia measuring modesty norms, mother–daughter communication, and menstrual readiness. Structural equation modeling assessed direct, indirect (mediation), and moderation effects. The qualitative phase included 20 in-depth interviews analyzed using Colaizzi's phenomenological approach to deepen interpretation of quantitative findings.

**Results:**

Islamic modesty norms were negatively associated with menstrual readiness (*β* = −0.21, *p* < 0.001), whereas mother–daughter communication demonstrated a strong positive association (*β* = 0.43, *p* < 0.001). Communication partially mediated the relationship between modesty norms and readiness (indirect *β* = −0.14, *p* < 0.001). Moderation analysis indicated that high modesty norms weakened the positive effect of communication. Qualitative findings showed menarche was often experienced as silent and private, with communication focused on rules rather than emotional support.

**Conclusion:**

Menarche is experienced as a culturally patterned silent transition shaped by modesty norms and relational communication. Strengthening emotionally supportive communication within Islamic values is essential to enhance menstrual readiness and reduce stigma in culturally responsive interventions.

## 1 Introduction

Menarche is a normal biological milestone, but for many girls it is not experienced as a simple or “ordinary” event. In many communities, the first menstruation happens quietly, with limited preparation, and sometimes with fear or embarrassment. Evidence from low- and middle-income countries (LMICs) shows that adolescent girls often reach menarche without enough knowledge, with many misconceptions, and with little practical guidance on how to manage menstruation (1, 2). This situation is not only about the lack of information; menstruation is also strongly connected to cultural and religious meanings that shape how girls understand their bodies and how adults respond to their needs (1).

A growing body of studies also shows that negative experiences at menarche are common when menstruation is considered a taboo topic. Girls may feel ashamed, worried, or confused, especially when they have never discussed menstruation openly before the first bleeding. For example, Ethiopian school communities reported barriers to menstrual hygiene management that were not only related to facilities and resources, but also to social discomfort and stigma at school (3). In rural Gambia, menstruation was surrounded by secrecy and embarrassment, making it difficult for girls to ask parents or teachers for advice, even when some girls had heard about menstruation before menarche (4). Similar patterns were reported in rural Tamil Nadu, where menarche was described not only as a biological transition but also as a moment when cultural codes and gender norms become stronger and shape how girls cope both at home and at school (5). In settings with social and structural constraints, such as refugee camps, menstrual preparedness can be even more difficult; girls, mothers, and teachers may have different views on when menstruation education should start, and many girls still enter menarche without adequate preparation (6). Recent qualitative work in Pakistan also highlights that first menstruation can be experienced as secretive and isolating because girls are given little prior information, while social expectations and restrictions become stronger at this time (7).

In current scholarship, menstruation is increasingly discussed as “menstrual health,” meaning it is not only about hygiene practices. Menstrual health includes knowledge, emotional well-being, supportive relationships, autonomy, and an enabling environment that allows girls to manage menstruation with dignity (8). This perspective fits with the idea of menstrual readiness, where girls need more than basic knowledge; they also need confidence, practical skills, and access to trusted support when they face menarche (1, 2). Research has also developed better measurement tools, such as the Menstrual Practice Needs Scale (MPNS-36), which helps capture how different needs during menstruation are met or not met, including the social and environmental aspects (9). At the broader level, improving menstrual health is also linked to education, gender equality, health, and other development goals, which shows why this topic is important beyond the individual experience (10).

One key factor shaping menstrual readiness is mother–daughter communication. In many LMIC settings, mothers are the main source of menstrual information, but mothers may not always have enough knowledge or feel comfortable to discuss menstruation in detail (1, 2). Girls themselves often report that they feel afraid or confused because explanations from adults come too late or are incomplete, and they may rely on peers instead (11). Studies on reproductive health communication also show that communication is influenced by comfort and family relationships, and the overall status of mother–daughter communication is often not optimal (12). Importantly, communication is not only about whether mothers talk or not; it is also about what kind of messages are delivered. For example, in Ghana, menstruation discussions were rare, and when they happened they often shifted to warnings about pregnancy and boys, which can make menstruation feel like a moral “risk” rather than a normal body process (13). Quantitative findings also support the value of good communication, showing that better mother–daughter communication is associated with more positive menstrual attitudes (14), and stronger mother–daughter attachment is linked with lower menstrual distress (15).

At the same time, menstruation is also regulated by restrictions and stigma across sociocultural and religious contexts. A mixed qualitative and quantitative study among Tanzanian girls showed that religion-based menstrual restrictions were common and could contribute to menstrual stigma, although stigma was also shaped by wider community and school contexts (16). This is very relevant for Muslim-majority settings, where modesty norms can strongly influence how girls manage bodily changes and how families discuss sensitive topics. Research on religion and intimate health suggests that religion should not be treated only as a demographic variable, because religious values can shape the acceptability of discussing intimate health topics and the way information is accessed (17). Based on this perspective, menarche can become a “silent transition” when modesty norms encourage concealment, limit open discussion, and reduce opportunities for girls to receive early preparation and emotional reassurance.

Indonesia, especially rural Muslim-majority areas, is an important context for this issue. Some Indonesian studies already show that maternal communication and support are associated with readiness to face menarche (18). However, not all findings are consistent; for example, one study in Jambi found no significant relationship between readiness and some family-related factors such as closeness and parenting style, suggesting that readiness pathways may differ across communities and measures (19). Another Indonesian study also highlights ongoing needs in menstrual hygiene determinants among adolescent schoolgirls (20). In addition, there are growing culturally grounded programs in Islamic educational settings. A mixed-method study in an Islamic boarding school in Yogyakarta showed that training interventions can improve hygiene practices (21). Community education in pesantren settings also reported increased knowledge related to menstrual myths, pain management, and the role of boys and men in menstruation discourse (22). Moreover, sharia-based menstrual education has been implemented to improve adolescent behavior during menstruation, indicating that faith-aligned approaches can be acceptable and potentially effective (23). However, most local initiatives focus on menstruation management after menarche, while the earlier stage, how girls are prepared before the first bleeding still needs more attention.

Despite increasing attention to menstrual health, little empirical work has simultaneously examined religious modesty norms, family communication dynamics, and menstrual readiness using a mixed-methods approach. Understanding these interconnected pathways is essential because culturally embedded silence may limit emotional preparedness even when informational support is present. Therefore, investigating menarche as a culturally patterned silent transition provides a more comprehensive framework for designing culturally responsive interventions tailored to rural Muslim adolescents.

Therefore, a clear research gap remains. Global evidence confirms that girls in LMICs are often underprepared for menarche, but fewer studies specifically examine how Islamic modesty norms shape mother–daughter communication and create a culturally “silent” transition in rural Muslim-majority settings (1, 2, 17). Many studies highlight the importance of communication, but do not deeply explore how the content of messages (e.g., restrictions, warnings, secrecy) influences menstrual readiness and emotional experiences at menarche (12, 13). In Indonesia, the evidence base shows maternal support and communication are relevant, but findings are mixed and often not explained through a culturally grounded framework that considers modesty norms and rural context (18–20, 24). Addressing these gaps, this study aims to (1) examine how Islamic modesty norms shape adolescents' menarche-related silence and disclosure practices in rural settings, (2) assess the association between mother–daughter communication quality and adolescents' menstrual readiness before and around menarche, and (3) generate culturally grounded evidence to inform culturally safe nursing, school, and community interventions that strengthen menstrual readiness and reduce stigma among rural Indonesian adolescents.

This study was guided by an integrated socioecological and family communication framework. Drawing on the Social Ecological Model and Family Communication Theory, Islamic modesty norms were conceptualized as sociocultural influences operating at the community and family levels, shaping expectations regarding bodily privacy and acceptable discourse. Mother–daughter communication was positioned as an interpersonal mechanism through which these norms influence individual outcomes. Menstrual readiness was conceptualized as an individual-level outcome reflecting knowledge, emotional preparedness, and help-seeking confidence. Within this framework, stronger modesty norms were expected to constrain open communication, which in turn would reduce menstrual readiness, while supportive communication may buffer the negative influence of restrictive norms.

Based on this framework, the following hypotheses were proposed:

- H1: Stronger Islamic modesty norms are negatively associated with adolescents’ menstrual readiness.
- H2: Higher-quality mother–daughter communication is positively associated with adolescents’ menstrual readiness.
- H3: Mother–daughter communication mediates the relationship between Islamic modesty norms and menstrual readiness.
- H4: Islamic modesty norms moderate the association between mother–daughter communication and menstrual readiness, such that the positive effect of communication is weaker at higher levels of modesty norms.

## 2 Methods

### 2.1 Study design

This study employed a cross-sectional explanatory sequential mixed-methods design to examine associations between Islamic modesty norms, mother–daughter communication, and menstrual readiness at menarche among rural Indonesian adolescents. In the first (quantitative) phase, a school-based survey was conducted to test theoretically informed associations and pathways between study variables. In the second (qualitative) phase, in-depth interviews were undertaken to explore adolescents' lived experiences and to explain sociocultural mechanisms underlying the quantitative patterns. Integration of quantitative and qualitative findings occurred at the interpretation stage using joint displays to generate meta-inferences.

### 2.2 Setting and participants

The quantitative phase was conducted in rural Muslim-majority communities in Indonesia, involving adolescent girls enrolled in public junior high schools and Islamic educational institutions. Eligible participants were female adolescents aged 10–15 years, residing in rural areas, identifying as Muslim, and either pre-menarche or within 12 months post-menarche, to capture menstrual readiness during the early transition period. Additional inclusion criteria included the ability to read Bahasa Indonesia and provision of written adolescent assent together with parental or guardian consent. Adolescents with diagnosed cognitive impairments or acute medical conditions that prevented independent questionnaire completion were excluded.

A multistage cluster sampling approach was applied in the quantitative phase. First, rural districts were purposively selected based on demographic and educational characteristics. Second, schools within each district were randomly selected. Third, eligible students were randomly selected from class lists provided by participating schools. Sample size was estimated *a priori* using G*Power based on multiple linear regression (fixed model, *R*^2^ deviation from zero) to approximate the requirements of the primary multivariable and latent-variable analyses. Assuming a small-to-moderate effect size (*f*^2^ = 0.08), a significance level of 0.05, statistical power of 0.95, and 8–10 predictors (Islamic modesty norms, mother–daughter communication, their interaction, and sociodemographic covariates), the minimum required sample size was calculated. To support complex analyses, including mediation and moderation testing, structural equation modeling, and adjustment for school-level clustering, the sample size was inflated to account for design effects and potential non-response, resulting in a target sample of approximately 350–450 participants.

Following preliminary quantitative analysis, the qualitative phase was conducted to explain and contextualize the quantitative findings. Participants were purposively selected from the quantitative sample using maximum variation sampling, based on levels of Islamic modesty norms, mother–daughter communication quality, menstrual readiness, and menarche status (pre-menarche vs. early post-menarche). A total of 20 in-depth interviews were conducted using a semi-structured interview guide exploring experiences of learning about menstruation, communication practices with mothers, perceived norms of Islamic modesty, emotional responses at menarche, and perceived support needs. The sample size for the qualitative phase was determined based on information power and data saturation, defined as the point at which no new substantive themes emerged from successive interviews. Saturation was reached by the twentieth interview, at which stage thematic patterns were well developed and conceptually coherent across participant profiles. The multistage cluster sampling procedure involved three rural districts in Southeast Sulawesi Province. Within each district, two to three schools were randomly selected, resulting in a total of eight participating schools. Approximately 45–55 eligible students were recruited from each school, yielding a balanced distribution of participants across clusters. This sampling structure justified the application of school-level clustering correction in the SEM analysis.

### 2.3 Measures and interview guideline

#### 2.3.1 Islamic modesty norms

Content validity of all adapted instruments was evaluated using a modified Delphi technique involving five experts in adolescent health nursing, reproductive health, and Islamic cultural studies. Experts rated item relevance, clarity, and cultural appropriateness using a 4-point scale. The Content Validity Index (CVI) was calculated, and items with I-CVI below 0.78 were revised. The overall scale-level CVI values ranged from 0.88 to 0.94, indicating satisfactory content validity prior to field testing. In the current sample, the adapted scale demonstrated acceptable internal consistency (Cronbach's *α* = 0.83; McDonald's *ω* = 0.88). Construct validity was evaluated using confirmatory factor analysis (CFA) prior to hypothesis testing. Scale scores were calculated as the mean of all items, yielding a possible range of 1–5. Confirmatory factor analysis demonstrated acceptable model fit (CFI = 0.94, TLI = 0.92, RMSEA = 0.05, SRMR = 0.04).

The original items were adapted for age-appropriateness through cognitive interviews with five adolescent girls aged 10–15 years during pilot testing. Minor wording modifications were made to simplify language and ensure comprehension while maintaining conceptual equivalence. These adaptations aimed to enhance cultural and developmental suitability for rural Indonesian adolescents.

#### Mother–daughter communication

Mother–daughter communication regarding menstruation was assessed using a menstruation-specific communication scale adapted from established parent–adolescent communication and reproductive health communication frameworks, including those proposed by Jaccard et al. and subsequent adolescent reproductive health studies. The adapted scale comprised 12 items, rated on a 5-point Likert scale (1 = strongly disagree to 5 = strongly agree), capturing key domains such as timing of communication, openness, emotional tone, practical guidance, and restriction-based or normative messaging related to menstruation. Higher scores indicate more supportive and comprehensive communication. As no standardized Indonesian version was available, the instrument underwent contextual adaptation and psychometric evaluation in this study. The scale demonstrated good internal consistency in the present sample (Cronbach's *α* = 0.81; McDonald's *ω* = 0.80). The CFA model showed acceptable fit indices (CFI = 0.93, TLI = 0.91, RMSEA = 0.06, SRMR = 0.05). Factor structure was examined using confirmatory factor analysis. Final scores were computed as the mean of item responses, with a possible range of 1–5.

#### Menstrual readiness

Menstrual readiness at menarche, the primary outcome variable, was measured using a multidimensional Menstrual Readiness Questionnaire, adapted from prior instruments assessing preparedness for menarche among adolescent girls (25). The questionnaire consisted of 16 items rated on a 5-point Likert scale, assessing four domains: anticipatory knowledge, practical preparedness, emotional confidence, and help-seeking readiness. Item scores were averaged to generate a total readiness score, with higher scores indicating greater menstrual readiness and a possible range of 1–5. Previous studies have reported acceptable reliability for similar instruments (Cronbach's *α* range = 0.78–0.88). In the present study, the adapted questionnaire demonstrated strong internal consistency (Cronbach's *α* = 0.86; McDonald's *ω* = 0.84). Construct validity was evaluated using confirmatory factor analysis, and the final model demonstrated acceptable fit indices. The final CFA model demonstrated good fit (CFI = 0.95, TLI = 0.93, RMSEA = 0.05, SRMR = 0.04).

#### Covariates

Covariates included age, school grade, school type (public vs. Islamic), maternal education, household socioeconomic proxy, menarche status (pre-menarche vs. early post-menarche), and access to private water, sanitation, and hygiene facilities at home and school. Menarche status (pre-menarche vs. early post-menarche) was included as a covariate to account for potential differences in experiential knowledge and emotional preparedness.

#### Interview guideline

A semi-structured interview guide was used to explore adolescents' experiences of menarche and the sociocultural factors shaping menstrual readiness. The guide examined how participants first learned about menstruation, the timing and content of mother–daughter communication, and perceived boundaries related to Islamic modesty norms. Interviews also explored emotional responses before and at menarche, including feelings of preparedness, fear, shame, or confidence, and how communication practices influenced these experiences. Participants were invited to describe coping strategies, support needs, and their views on what constituted supportive and culturally appropriate communication about menstruation.

#### Interview schedule

The semi-structured interview schedule was developed based on the study objectives and existing literature on menstrual readiness, modesty norms, and parent–adolescent communication. The guide included five core domains:

1. Initial learning about menstruation
  - “When did you first hear about menstruation?”
  - “Who told you about it?”
2. Mother–daughter communication
  - “What did your mother explain about menstruation?”
  - “How comfortable did you feel asking questions?”
3. Islamic modesty norms
  - “Are there rules about discussing menstruation in your family?”
  - “How do these rules influence your willingness to talk?”
4. Emotional responses around menarche
  - “How did you feel when you first experienced menstruation?”
  - “Did anyone provide emotional support?”
5. Support needs and recommendations
  - “What kind of communication would help girls feel more prepared?”
  - “How can guidance respect Islamic values?”

The interview guide was pilot-tested with three adolescents to ensure clarity and cultural appropriateness before data collection.

### 2.4 Procedure

Following institutional ethical approval, permissions were obtained from local education authorities and school administrators prior to data collection. Information sheets were provided to parents or guardians and adolescents, and written parental consent together with adolescent assent were obtained before participation. The quantitative phase was conducted first using self-administered questionnaires completed in supervised school settings to ensure privacy and comprehension. Upon completion of the survey, participants received brief, age-appropriate menstrual health information and referral pathways for adolescent health services if needed. The qualitative phase followed preliminary quantitative analysis, with purposively selected participants invited for in-depth interviews conducted in private settings within schools or community facilities. Interviews were audio-recorded with permission and transcribed verbatim, and participants were given the opportunity to provide feedback or clarification on summarized interpretations.

#### Data analysis

Quantitative data were analyzed using SPSS version 29 for several sequential stages. First, descriptive statistics were used to summarize participants' demographic characteristics, including age group, menarche status, school type, mother's education, and household WASH access, using frequencies, percentages, means, and standard deviations (Table 1). Second, univariate descriptive analyses were conducted to estimate central tendency and dispersion for the main study variables, namely Islamic modesty norms, mother–daughter communication, and menstrual readiness using mean scores and standard deviations on their respective Likert scales (Table 2). Third, bivariate associations among the key variables were examined using Pearson's correlation coefficients to assess the direction and strength of relationships between Islamic modesty norms, mother–daughter communication, and menstrual readiness (Table 3). These correlation analyses provided preliminary evidence to inform subsequent multivariable and structural modeling. Following these preliminary analyses, structural equation modeling (SEM) was conducted in Mplus version 8.10. Hypothesis testing in the structural model was conducted using maximum likelihood estimation with robust standard errors (MLR) based on scale scores, enabling the estimation of bootstrapped confidence intervals for direct, indirect, and interaction effects under school-level clustering. All statistical tests were two-tailed, with a significance level set at *p* < 0.05.

**Table 1:** Demographic characteristics of participants (*N* = 386).

Variable	n	%
Age group (years)		
10–11	92	23.8
12–13	168	43.5
14–15	126	32.6
Menarche status		
Pre-menarche	182	47.2
≤12 months post-menarche	204	52.8
School type		
Public junior high school	221	57.3
Islamic school/pesantren	165	42.7
Mother's education		
Primary or less	179	46.4
Secondary	141	36.5
Higher education	66	17.1
Private WASH access at home		
Yes	267	69.2
No/limited	119	30.8

**Table 2:** Descriptive statistics of study variables.

Variable	Mean ± SD	Range
Islamic modesty norms	3.74 ± 0.62	1–5
Mother–daughter communication	3.21 ± 0.69	1–5
Menstrual readiness (total score)	3.09 ± 0.73	1–5

**Table 3:** Correlation matrix between islamic modesty norms, mother–daughter communication, and menstrual readiness (*N* = 386).

Variable	1	2	3
1. Islamic modesty norms	1		
2. Mother–daughter communication	−0.34***	1	
3. Menstrual readiness	−0.29***	0.48***	1

Values represent Pearson's correlation coefficients.

**p < 0.001.

Qualitative data were analyzed using Colaizzi's phenomenological method, which provides a systematic and rigorous approach for examining lived experiences. Analysis followed Colaizzi's six-step procedure: repeated reading of transcripts for familiarization, extraction of significant statements, formulation of meanings, clustering of meanings into themes, development of an exhaustive description, and validation of findings through participant feedback to enhance credibility (28, 29). Integration of quantitative and qualitative findings occurred at the interpretation stage using joint displays, enabling explanation of how Islamic modesty norms and mother–daughter communication patterns co-occurred with levels of menstrual readiness.

Credibility was enhanced through multiple strategies. Prolonged engagement with the data was ensured through repeated reading of transcripts during analysis, and analytic decisions were documented using reflexive memos to support transparency. Peer debriefing was conducted among the research team to discuss coding decisions and emerging themes, reducing individual interpretive bias. In addition, member checking was undertaken by sharing summarized interpretations with selected participants to confirm the accuracy and resonance of the findings with their lived experiences.

### 2.5 Ethical consideration

This study was conducted in accordance with the ethical principles of the Declaration of Helsinki and received ethical approval from the Health Research Ethics Committee of STIKep PPNI Jawa Barat, Indonesia (approval number: 098ETIK). Participation was entirely voluntary, and written parental or guardian consent together with adolescent assent were obtained prior to data collection. All data were anonymized, stored securely, and accessible only to the research team. Participants were informed of their right to withdraw from the study at any stage without any consequences. Given the sensitive nature of menstruation-related discussions, all interviews were conducted by trained female researchers, and procedures were implemented to minimize discomfort and ensure a culturally respectful and supportive research environment.

## 3 Results

A total of 386 adolescent girls participated in the quantitative phase (response rate: 91.2%). Participants had a mean age of 12.9 ± 1.2 years (range 10–15 years). Slightly more than half of the participants were post-menarche but within 12 months of first menstruation (Table 1).

Table 2 indicates that participants demonstrated a moderately high endorsement of Islamic modesty norms (mean = 3.74 ± 0.62 on a 5-point scale), suggesting that modesty-related beliefs regarding bodily privacy and menstrual discretion are widely internalized among rural Indonesian adolescents. In contrast, mother–daughter communication quality was moderate (mean = 3.21 ± 0.69), indicating variability in the openness, emotional tone, and practical guidance provided by mothers about menstruation. The mean menstrual readiness score was 3.09 ± 0.73, reflecting a moderate level of preparedness overall.

The correlation matrix demonstrates clear and theoretically coherent relationships among the key study variables (Table 3). Islamic modesty norms were moderately and negatively correlated with mother–daughter communication (*r* = −0.34, *p* < 0.001), indicating that stronger endorsement of modesty norms was associated with lower levels of open and supportive menstrual communication. Menstrual readiness showed a moderate negative correlation with Islamic modesty norms (*r* = −0.29, *p* < 0.001), suggesting that adolescents who strongly internalized modesty-related restrictions tended to be less prepared for menarche. In contrast, mother–daughter communication was moderately to strongly positively correlated with menstrual readiness (*r* = 0.48, *p* < 0.001), indicating that higher-quality communication was associated with greater preparedness.

Islamic modesty norms showed a significant negative association with menstrual readiness (*β* = −0.21, 95% CI: −0.31 to −0.11, *p* < 0.001), indicating that stronger endorsement of modesty norms was linked to lower levels of preparedness. In contrast, mother–daughter communication demonstrated a strong positive association with menstrual readiness (*β* = 0.43, 95% CI: 0.31–0.55, *p* < 0.001) and emerged as the largest effect in the model. Age was positively associated with readiness (*β* = 0.12, 95% CI: 0.04–0.20, *p* = 0.003), whereas mother's education showed a small and non-significant association (*β* = 0.08, 95% CI: −0.01 to 0.18, *p* = 0.091).

Mediation analysis indicated that mother–daughter communication partially accounted for the association between Islamic modesty norms and menstrual readiness (indirect *β* = −0.14, 95% CI: −0.21 to −0.08, *p* < 0.001), suggesting that stronger modesty norms were associated with poorer communication quality, which in turn related to lower readiness. The significant interaction between modesty norms and communication (*β* = −0.17, 95% CI: −0.29 to −0.05, *p* = 0.004) indicates that the positive association between communication and menstrual readiness was attenuated at higher levels of Islamic modesty norms (Table 4).

**Table 4:** Structural equation model results for menstrual readiness: direct, indirect (mediation), and moderation effects.

Path	Standardized *β*	SE	95% CI	*p*-value
Direct effects				
Islamic modesty norms → Menstrual readiness	−0.21	0.05	−0.31 to −0.11	<0.001
Mother–daughter communication → Menstrual readiness	0.43	0.06	0.31 to 0.55	<0.001
Age → Menstrual readiness	0.12	0.04	0.04 to 0.20	0.003
Mother's education → Menstrual readiness	0.08	0.05	−0.01 to 0.18	0.091
Indirect (mediation) effect				
Islamic modesty norms → Communication → Readiness	−0.14	—	−0.21 to −0.08	<0.001
Moderation effect				
Modesty norms × Communication → Readiness	−0.17	0.06	−0.29 to −0.05	0.004

Standardized coefficients (β) are reported. Structural equation modeling was estimated in Mplus using MLR with TYPE = COMPLEX. Bootstrapped 95% confidence intervals (5,000 resamples) are reported for direct, indirect, and interaction effects. SE = robust standard error; CI = confidence interval.

The qualitative findings show that menarche is commonly experienced as a quiet and deeply personal moment, rather than something openly discussed or emotionally supported (Table 5). Many adolescents described menarche as something that should be handled discreetly, where silence is seen as part of being a “good” and modest Muslim girl. In this context, not talking about menstruation is not simply a lack of information, but a learned form of moral behavior. Communication with mothers was generally present but practical in nature. Most girls received instructions about what to do during menstruation, especially related to cleanliness and religious rules. However, emotional aspects, such as fear, confusion, or anxiety were rarely addressed. As a result, several participants felt that although they “knew the rules,” they were still emotionally unprepared when menarche occurred. At the same time, adolescents clearly expressed a desire for supportive communication that remains respectful of Islamic values. They did not question religious teachings; instead, they wanted explanations delivered in a calmer and more caring way. Many girls hoped for a safe space where they could ask questions without feeling embarrassed or judged, showing that modesty and preparedness are not seen as opposing values.

**Table 5:** Themes, categories, codes, and representative quotes (*N* = 20).

Theme	Category	Code	Representative quotes
Theme 1: Menarche as a Silent and Private Transition	Menstruation as a private matter	Menarche should be hidden	“When I first got my period, my mother told me not to let anyone know, especially people outside the family.” (P7) “Menstruation is something private. We are taught to keep it to ourselves.” (P12)
		Avoid public disclosure	“I didn’t tell my friends because it felt embarrassing to talk about.” (P3) “I stayed quiet and tried to manage it alone at school.” (P15)
	Silence as moral discipline	Silence equals modesty	“Being quiet about menstruation means you are a polite girl.” (P1) “If we talk too much about it, people may think we are not modest.” (P9)
		Fear of being judged	“I was afraid people would judge me if they knew.” (P6) “Talking about menstruation feels like exposing yourself.” (P18)
Theme 2: Communication Framed as Instruction, Not Emotional Support	Rule-based communication	Instruction without explanation	“My mother only told me what to do, like how to wash and change pads.” (P4) “She said this is what girls must do, without explaining why.” (P10)
		Focus on restrictions	“She warned me not to pray and not to talk about it.” (P14) “Most of the advice was about what is allowed and what is forbidden.” (P19)
	Limited emotional dialogue	Emotional needs not discussed	“I knew what menstruation was, but I was still scared.” (P8) “No one asked how I felt when it happened.” (P16)
		Feeling emotionally unprepared	“Even though I had information, I felt confused and nervous.” (P2) “I understood the rules, but not my feelings.” (P11)
Theme 3: Seeking Readiness within Religious Boundaries	Desire for safe communication	Wanting permission to ask	“I wanted to ask questions, but I was not sure if it was okay.” (P5) “Sometimes I had questions, but I kept them to myself.” (P17)
		Need for trusted guidance	“I feel safer when my mother explains calmly.” (P13) “I wish there was someone who could explain without judging.” (P20)
	Integrating religion and support	Religion as guidance, not fear	“Religion should help us understand, not make us afraid.” (P6) “I want explanations that follow Islam but are kind.” (P9)
		Balancing modesty and readiness	“We can still be modest and learn properly.” (P1) “Being a good Muslim girl doesn’t mean being unprepared.” (P12)

Integration of quantitative and qualitative findings revealed a clear convergence between the two phases. Quantitatively, Islamic modesty norms were negatively associated with menstrual readiness, both directly and indirectly through reduced mother–daughter communication. Mother–daughter communication emerged as the strongest positive predictor of readiness and buffered the negative influence of modesty norms. Qualitative narratives provided contextual explanations for these statistical relationships. Adolescents described silence around menstruation as a moral expectation linked to modesty, while communication with mothers was often rule-based and focused on religious obligations rather than emotional reassurance (Table 6).

**Table 6:** Joint display integrating quantitative and qualitative findings.

Quantitative findings	Qualitative findings	Integrated interpretation
Islamic modesty norms were negatively associated with menstrual readiness (*β* = −0.21, *p* < 0.001).	Adolescents described menstruation as something that should be hidden and not discussed openly to maintain modesty.	Strong modesty norms promote silence, which limits emotional and practical preparation for menarche.
Mother–daughter communication showed a strong positive association with readiness (*β* = 0.43, *p* < 0.001).	Mothers were described as giving practical instructions but rarely discussing emotions or fears.	Communication improves readiness, but its effectiveness depends on emotional as well as informational content.
Communication partially mediated the effect of modesty norms on readiness (indirect *β* = −0.14, *p* < 0.001).	Silence and rule-based messaging reduced opportunities for questions and reassurance.	Modesty norms influence readiness primarily by shaping how and whether communication occurs.
High-quality communication buffered the negative effect of modesty norms (interaction *β* = −0.17, *p* = 0.004).	Adolescents expressed a desire for guidance that respects Islamic values while allowing supportive dialogue.	Supportive communication within religious boundaries can mitigate the restrictive effects of modesty norms.

## 4 Discussion

The findings support the hypothesized socioecological communication pathway, in which cultural norms influence individual readiness through interpersonal communication mechanisms. This mixed-methods study provides robust evidence that menstrual readiness among rural Indonesian Muslim adolescents is shaped by the interplay between Islamic modesty norms and mother–daughter communication. Quantitatively, stronger endorsement of Islamic modesty norms was associated with lower menstrual readiness, both directly and indirectly through reduced communication quality. In contrast, mother–daughter communication emerged as the strongest positive predictor of readiness, highlighting the central role of relational processes in shaping adolescents' preparedness for menarche.

The negative association between modesty norms and readiness aligns with global evidence showing that sociocultural norms emphasizing silence and bodily privacy often limit girls' access to timely, comprehensive menstrual information (2, 9). However, the present findings extend prior work by demonstrating that modesty norms do not operate in isolation; rather, they influence readiness primarily by shaping how, when, and whether communication occurs within families. This is supported by the significant mediation effect, indicating that poorer communication partially explains the link between modesty norms and lower readiness.

Qualitative findings provided critical contextual insight into these statistical patterns. Adolescents consistently described menarche as a private and silent transition, where not talking about menstruation was understood as a form of moral discipline and proper religious behavior. Similar narratives of silence as virtue have been reported in qualitative studies from Muslim-majority and other conservative contexts, including Bangladesh, Ethiopia, and Tanzania, where menstruation is framed as something to be managed discreetly to maintain social respectability (3, 12, 13). In the Indonesian rural setting, this silence was not experienced as neglect, but as a learned cultural expectation.

While most participants reported receiving some information from their mothers, communication was predominantly instructional and rule-based, focusing on hygiene practices and religious restrictions. Emotional aspects, such as fear, confusion, or reassurance were rarely addressed. This pattern mirrors findings from studies in Ghana, India, and Pakistan, where mothers often act as primary information sources but avoid emotional dialogue due to discomfort, limited knowledge, or normative expectations (5, 7, 13). Importantly, although adolescents often felt “informed,” many still described feeling emotionally unprepared at menarche. This distinction between informational readiness and emotional readiness has been increasingly emphasized in menstrual health literature, which argues that knowledge alone is insufficient without emotional support and opportunities for safe questioning (9, 10). The strong positive association between communication quality and readiness in this study reinforces the idea that supportive, open communication rather than instruction alone is essential for fostering confidence and emotional preparedness.

The observed moderation effect further suggests that even high-quality communication may be constrained in contexts where modesty norms are strongly internalized. At higher levels of modesty endorsement, the positive impact of communication on readiness was attenuated, indicating that cultural norms can limit how effectively information and support are conveyed. This finding resonates with socio-cultural analyses showing that deeply embedded norms may shape not only whether communication occurs, but also its tone, depth, and emotional safety (17, 26).

By integrating quantitative and qualitative findings, this study conceptualizes menarche as a culturally patterned silent transition in rural Indonesian Muslim communities. Silence around menstruation is not merely a communication gap, but a meaningful social practice tied to religious identity, femininity, and moral conduct. Adolescents did not reject Islamic values; rather, they expressed a desire for guidance that remains respectful of religious norms while allowing emotional reassurance and clarity. This nuanced understanding challenges simplistic assumptions that religious norms are inherently harmful to menstrual preparedness. Instead, the findings suggest that the issue lies in how these norms are enacted in everyday communication. Similar conclusions have been drawn in recent work emphasizing that menstrual health interventions must engage with, rather than dismiss, religious and cultural frameworks to be effective and culturally safe (8).

Recent adolescent health frameworks emphasize emotionally supportive communication as a protective factor for developmental transitions. Emerging guidance from psychological and adolescent health literature highlights the importance of culturally sensitive dialogue that integrates emotional validation with accurate information. The present findings support this perspective and suggest testable hypotheses for future studies, such as examining whether structured mother-focused communication training improves menstrual readiness and reduces anxiety during menarche. The sequential explanatory mixed-methods design strengthened validity by integrating statistical relationships with lived experiences, allowing deeper interpretation of culturally shaped menstrual readiness.

## 5 Implications

The findings have several important implications for nursing practice, school health programs, and community-based interventions. First, nurses and school health professionals working with adolescents should recognize that menstrual readiness is shaped by both cultural norms and family communication patterns. Education efforts that focus solely on biological information may fail to address emotional needs, particularly in contexts where open discussion is constrained by modesty norms. Second, maternal education initiatives should move beyond rule-based messaging to include guidance on emotionally supportive communication. Training mothers to provide calm explanations, validate emotions, and invite questions while still respecting Islamic values may substantially improve adolescents' readiness. Evidence from intervention studies in Indonesia and other low- and middle-income settings suggests that culturally adapted, family-centered education can improve menstrual knowledge and practices without challenging core religious beliefs (21). Third, collaboration with religious leaders and Islamic schools may help reframe menstrual education as compatible with modesty and faith. Adolescents in this study explicitly expressed that religion should serve as guidance rather than fear. Integrating menstrual health messages into religious education, framed around care, dignity, and wellbeing, may reduce stigma and silence while maintaining cultural legitimacy (27).

## 6 Study limitations

Several limitations should be considered when interpreting the findings. First, the cross-sectional design of the quantitative phase limits causal inference; longitudinal studies are needed to examine how communication patterns and readiness evolve over time. Second, data relied on self-reported measures, which may be influenced by social desirability, particularly given the sensitivity of menstruation-related topics. Third, the study focused on rural Muslim-majority communities, which may limit generalizability to urban settings or adolescents from different religious backgrounds. Finally, although the mixed-methods design strengthened interpretation, qualitative interviews were conducted with a relatively small sample and may not capture all possible experiences.

Additionally, common method bias may be present because all variables were measured using self-report questionnaires in a single survey administration. Harman's single-factor test indicated that the first factor accounted for 32.4% of the variance, below the recommended threshold of 50%, suggesting that common method bias was unlikely to substantially influence the findings.

## 7 Conclusion

This study demonstrates that menstrual readiness among rural Indonesian Muslim adolescents is shaped by a complex interaction between Islamic modesty norms and mother–daughter communication. Strong modesty norms are associated with lower readiness primarily by constraining open and emotionally supportive communication. At the same time, adolescents express a clear desire for guidance that respects religious values while providing reassurance and clarity. Framing menarche as a culturally patterned silent transition highlights the need for culturally safe, relationally focused interventions that support both mothers and daughters. Strengthening emotionally supportive communication within religious boundaries represents a promising pathway to improve menstrual readiness and reduce stigma among rural Indonesian adolescents.

## Data Availability

The datasets presented in this study can be found in online repositories. The names of the repository/repositories and accession number(s) can be found in the article/Supplementary Material.
